# Association of prosthetic angles of the Implant Supracrestal Complex with peri‐implant tissue mucositis

**DOI:** 10.1002/cre2.750

**Published:** 2023-05-17

**Authors:** Piboon Rungtanakiat, Natchaya Thitaphanich, Wareerat Chengprapakorn, Martin Janda, Mansuang Arksornnukit, Nikos Mattheos

**Affiliations:** ^1^ Department of Prosthodontics, Faculty of Dentistry Chulalongkorn University Bangkok Thailand; ^2^ Department of Prosthodontics, Faculty of Odontology Malmoe University Malmo Sweden; ^3^ Department of Oral and Maxillofacial Surgery, Faculty of Dentistry Chulalongkorn University Bangkok Thailand; ^4^ Department of Dental Medicine Karolinska Institute Stockholm Sweden

**Keywords:** dental implants, emergence angle, Implant Supracrestal Complex, peri‐implant tissue

## Abstract

**Objectives:**

The aim of this study was to investigate the association of the Mucosal Emergence Angle (MEA) with peri‐implant tissue mucositis.

**Material and Methods:**

Forty‐seven patients with 103 posterior bone level implants underwent clinical and radiographic examination. Three‐dimensional data from Cone Bean Computer Tomography and Optica Scan were transposed. Three angles were defined: MEA, Deep Angle (DA) and Total Angle (TA) and measured at six sites for each implant.

**Results:**

There was a significant correlation between MEA and Bleeding on Probing for all sites with an overall odds ratio of odd ratio 1.07 (95% confidence interval [CI] 1.05–1.09, *p* < 0.001). Sites with MEA ≥ 30°, 40°, 50°, 60°, and 70° had a higher risk for bleeding with an odds ratio of 3.1, 5, 7.5, 11.4 and 33.55, respectively. When all 6 sites of an implant prostheses had MEA ≥ 40°, the risk of having bleeding at all 6 sites was 9.5 times higher (95% CI 1.70–52.97, *p* = 0.010).

**Conclusions:**

Maintaining MEA no wider than 30°−40° is advisable, while the aim should be to keep this angle as narrow as clinically feasible. Registered in Thai Clinical Trials Registry: http://www.thaiclinicaltrials.org/show/TCTR20220204002.

## 1 INTRODUCTION

There is an emerging body of evidence pointing towards significant interrelations between the condition of the peri‐implant tissue and the design of the prosthetic elements (Mattheos, Janda, et al., 2021). Three cross‐sectional studies (Katafuchi et al., 2018; Majzoub et al., 2021; Yi et al., 2020) have suggested that prosthesis contour of more than 30° as it appears in periapical radiographs, is correlated with increased risk for peri‐implantitis in bone level implants. Two cross sectional studies which followed similar methodology did not confirm the correlation of wide contour with peri‐implantitis (Inoue et al., 2020, Lops et al., 2022). Furthermore, convexity of the prosthesis profile has been correlated with increased recession (Siegenthaler et al., 2022), marginal bone loss (Valente et al., 2020) and peri‐implantitis when combined with overcontouring of the prosthesis (Yi et al., 2020). At the same time, defining the complex three‐dimensional (3D) structure of the prosthesis contour solely by means of the 2‐dimensional interproximal projection on periapical radiographs might be insufficient. In addition, the Glossary of Prosthodontic Terms (2017) 9th Edition, as well as previous research (Yotnuengnit et al., 2008), defines Emergence Profile (EP) and Angle (EA) only in relation to the “circumscribed soft tissues,” which cannot be accounted for in periapical radiographs. Nevertheless, the findings of these studies collectively warrant further investigation with a precise and reproducible protocol.

The aim of the present cross‐sectional study was to investigate the association of the prosthesis angle at the point of emergence with peri‐implant tissue mucositis, by means of defining and reproducibly measuring on transposed 3D radiographic and optical imaging. In particular, the study defined and measured the angle of the prosthesis at the point of emergence through the soft tissue (Mucosal Emergence Angle [MEA]) and the angle of the abutment at the point ascending from the bone level (Deep Angle [DA]).

## 2 MATERIAL AND METHODS

This study was approved by the Ethics Committee at the Faculty of Dentistry, Chulalongkorn University (HREC‐DCU 2022‐024) and was registered at the Thai Clinical Trials Registry (TCTR20210709003). The study followed the STROBE statements.

### 2.1 Patient sample

Systemically healthy patients (ASA classification I and II) (Daniel et al., 2023) aged 20 years or older, having received implant therapy at the postgraduate clinics of Prosthodontics or the clinic of Implants and Aesthetics, Faculty of Dentistry, Chulalongkorn University and currently in maintenance for at least 3 months were considered for the study. Patients in regular maintenance visits between July 4 and November 30, 2022 were invited to participate in the study if the following conditions were met:

(1) Posterior Implant Supported Single Crown.
(2) Bone level implants.
(3) Implants restored with the use of an abutment.
(4) Prosthesis axis no more than 10° divergence from implant axis.
(5) At least 3 months postrestoration.

Exclusion Criteria were:

1. patients with systemic disease and/or other conditions under the classification ASA III
2. Female patients in pregnancy or lactation.

Upon informed consent, the patients received clinical and radiographic examination.

### 2.2 Clinical examination for implant site

**Figure 1:**
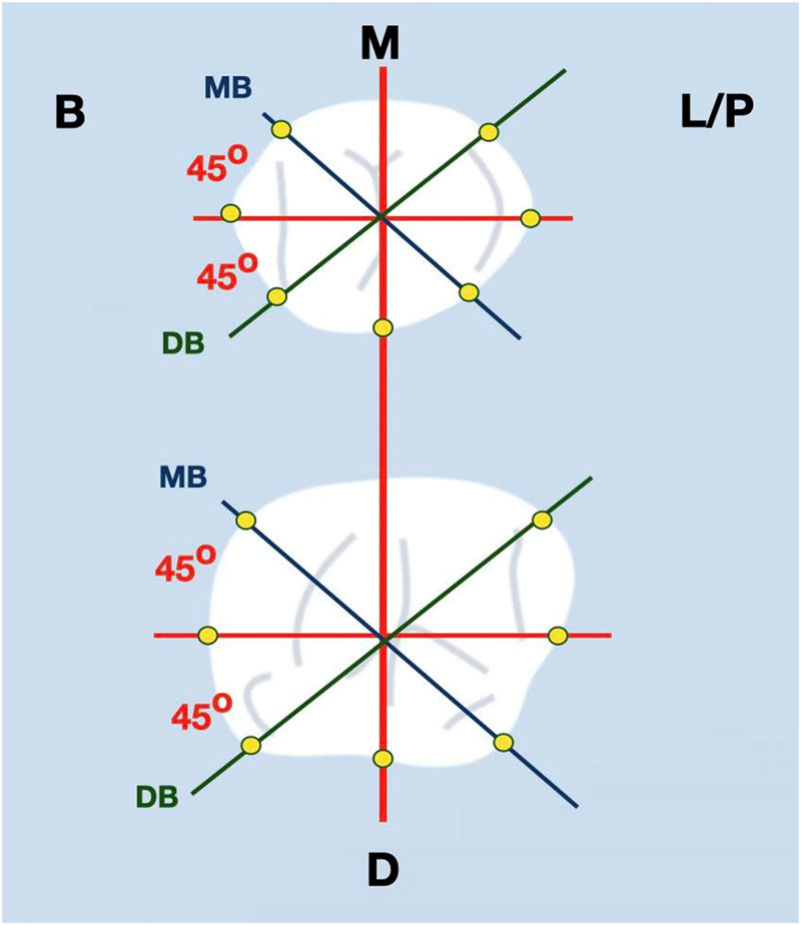
Six sites (Mesiobuccal MB, Midbuccal BB, Distobuccal DB and respective Midlingual/midpalatal LL, Distolingual DL and MesioLingual ML) were defined based on three planes for each implant crown. Plaque Index, Bleeding on Probing and Probing Depth was recorded at all six. Width of keratinized Mucosa was recorded only at the three buccal sites.

− Plaque index, dichotomous registration (O'Leary et al., 1972)
− Bleeding on probing (BoP), dichotomous registration
− Presence of suppuration, dichotomous registration
− Probing depth rounded to nearest mm at 6‐points per crown (Mesiobuccal BM, Midbuccal BB, Distobuccal BD and respective midlingual/midpalatal LM, Distolingual LD and MesioLingual LM) (Figure 1) with plastic periodontal probe (Hu‐Friedy).
− Keratinised Mucosa width in mm, at 3‐points per crown (buccal, buccal mesial, buccal distal)
− Clinical Photography (occlusal, buccal, and lingual views)
− Intraoral Scan with optical scanner (Trios 4, 3 Shape AS, Denmark). A Standard Tessellation Language (STL) file was exported for each patient. All clinical measurements were conducted by one expert examiner. Intrarater agreement was assessed by means of a calibration exercise measuring peri‐implant probing depth in four patients at two subsequent visits, 48 h apart. Intraclass correlation coefficient was ICC = 0.931 (95% CI: 0.840−0.970), *p* < 0.001).

In the cases where excessive contour of the prosthesis prevented insertion of the probe or compromised the ability to probe the sulcus in vertical direction, the probing depth was not recorded.

### 2.3 Radiographic examination

− Periapical radiograph, with long cone parallel technique
− Narrow field Cone Beam CT (3D X‐mind Trium de Götzen S.r.l.‐Acteon Group—7 mA, 70 kV, 63‐s exposure time, 0.15 × 0.15 × 0.15 mm voxel size, field of view 11 × 9 cm). A DICOM file was exported for each patient.

### 2.4 Measurements

Digital Imaging and Communications in Medicine (DICOM) file of CBCT was imported in the treatment planning software (coDiagnostiX version 9.7, Dental Wings Inc). Thereafter, and the intraoral optical scan (STL file) was imported through the standard software option of “alignment to another object” (DICOM). Three pairs of corresponding anatomic regions on natural teeth were defined on the intraoral scan and registration object (DICOM). Transposition of the STL file on the CBCT data was then conducted automatically by the software.

The implant axis and the perpendicular axis of the implant platform were defined. Thereafter, three Vertical Planes (parallel to Implant Axis) were identified: (Figure 1)

(i) Midline (buccolingual plane)
(ii) 45° mesiobuccal
(iii) 45° distobuccal.

**Figure 2:**
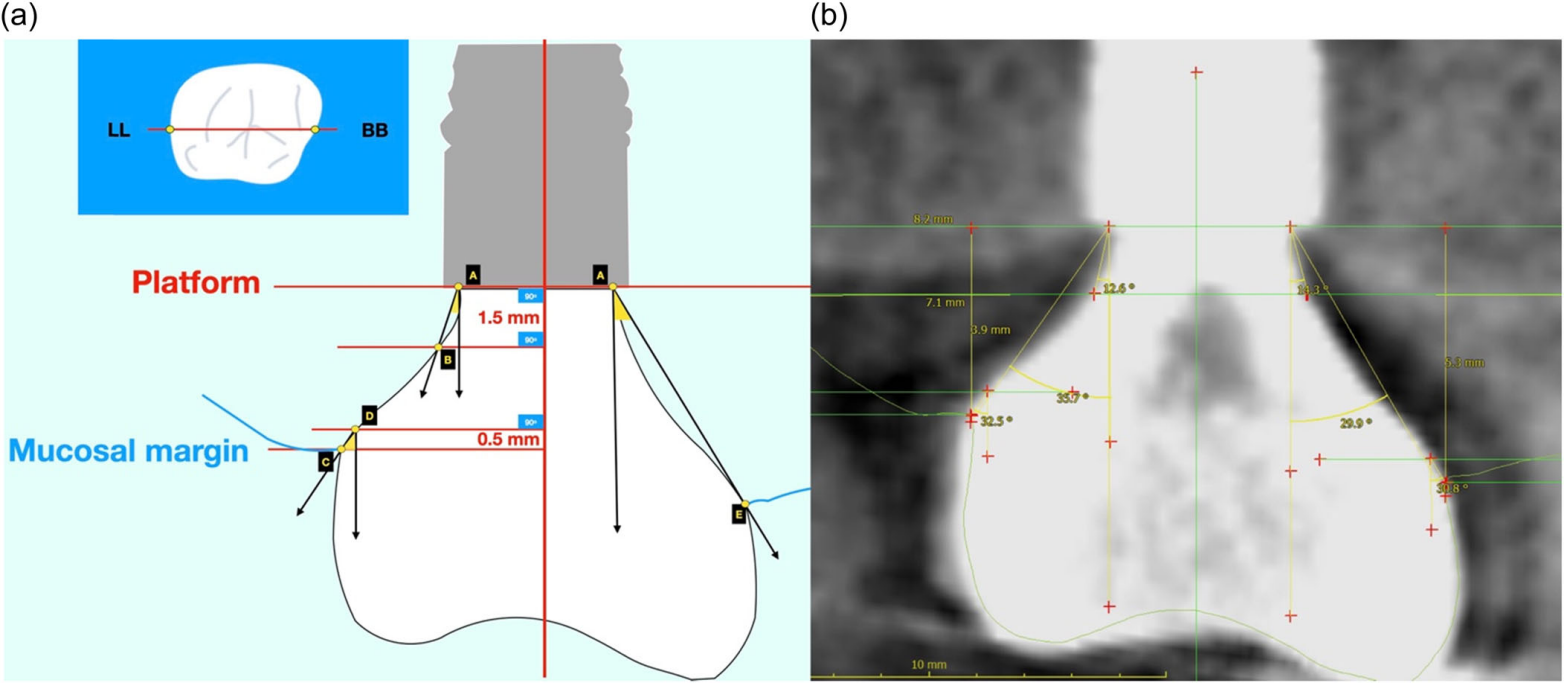
(a) Buccal—Palatal slice and definition of 4 horizontal planes perpendicular to implant axis, 5‐points (A‐E) and the respective 3 angles measured on each side (six sites per implant). (b) sample of actual radiographic measurement screen.

Each plane included two side views of the Implant Supracrestal Complex, so a total of six sites were identified for each implant (Figure 1). The following horizonal levels (perpendicular to the Implant Axis) were defined for each site: (Figure 2).

(i) Implant platform
(ii) 1.5 mm coronally of implant platform
(iii) Mucosal Margin
(iv) 0.5 mm apically of Mucosal Margin.

Thereafter, the following points were identified for each level:

A: Point of Implant abutment nearest to the implant platform.
B: Point of Implant abutment 1.5 mm coronally of point A (projected on Implant Axis).
C: Most Coronal point of soft tissue (Mucosal Margin).
D: Point of prosthesis 0.5 mm apically of Mucosal Margin (projected on Implant Axis).
E: Point of prosthesis at maximum convexity/prominence.

Three angles were defined as follows:

(i) Deep Angle (DA): angle of the abutment ascending directly from implant platform. Defined by the implant axis and the line connecting A−B.
(ii) Mucosal Emergence Angle (MEA): angle of the prosthesis emerging through the soft tissue. Defined by the implant axis and the line connecting C–D.
(iii) Total Contour Angle (CA): angle of the overall contour of the prosthesis. Defined by the implant axis and the line connecting A−E.

All radiographic measurements were conducted by two calibrated examiners, blinded to the clinical examination measurements (Figure 2). Inter‐rater agreement between the two examiners was assessed during a calibration exercise where the two examiners calculated independently the MEA in a set of 10 sections. The using intraclass correlation coefficient was 0.998 (0.995−0.999) *p* < .001.

### 2.5 Clinical diagnoses

Diagnosis at implant level was based on the condition of the peri‐implant tissue upon clinical and radiographical examination.

#### 2.5.1 Mucositis

Peri‐implant tissue inflammation was recorded by means of bleeding or suppuration on gentle probing with light pressure (Berglundh et al., 2018). Number of BoP sites out of six was reported for each implant. Mucositis at implant level was described by means of the extent of BoP (number of BoP sites out of six) as in Vianna et al. (2018) and Philip et al. (2022).

#### 2.5.2 Peri‐implantitis

Three case definitions were used for Peri‐implantitis:

(a) presence of bleeding and/or suppuration on gentle probing, presence of at least one site with Probing depth ≥ 6 mm and level of bone ≥ 3 mm apical of the most coronal portion of the intraosseous part of the implant, as seen in the periapical radiographs (day‐to‐day practice, Berglundh et al., 2018)
(b) presence of bleeding and/or suppuration on gentle probing combined with level of bone ≥ 3 mm apical of the most coronal portion of the intraosseous part of the implant, as seen in the periapical radiographs (epidemiological studies, Berglundh et al., 2018)
(c) the same as above with the bone level threshold set at ≥2 mm (Romandini et al., 2021). Periapical radiographs were inserted in Image J software (National Institute of Health) and marginal bone level at mesial and distal of the implant was calculated in relation to the implant platform after calibration using the implant length and width.

## 3 SAMPLE SIZE CALCULATION

The association of MEA and peri‐implant tissue inflammation has not been previously investigated, thus limiting the ability to use specific data for a sample size calculation. Previous studies (Yi et al., 2020), have established the threshold 30° for studying the impact of the CA and found difference in the prevalence of peri‐implantitis between 16% and 33%. Albeit not directly related measurements, assuming a minimum effect size of at least 25% to detect the difference in the prevalence of BoP (primary outcome of this study) between MEA ≥ 30 and MEA < 30, a sample size of 90 cases is required using a significant level of 0.05 (two‐sided) and study power of 80%. Adjusting the sample size by 10% for missing data, would result in a minimum sample size of 100 implants. The sample size calculation was based on the hypothesis tests for two independent proportions.

## 4 STATISTICAL ANALYSIS

Descriptive statistics were presented using mean, standard deviation (SD), median, interquartile range (IQR), and percentages as appropriate. Associations between angles and BoP were assessed using three‐level mixed models for logistic regression to account for multiple sites within implant and within patient by using implant level and patient level as random intercept. Logistic regression analysis was done for the implant level, to explore the association between BOP and the MEA ≥ 40° for all 6 sites. The generalized estimating equations (GEE) were performed to account for multiple implants within patients for the analysis at implant level. The association between the number of sites (0−6) with different cut points of the angles and number of bleeding sites at implant level, (0−6) was investigated using GEE with poisson family and log link, and the association between angles and peri‐implantitis (case definition) was investigated using GEE with binomial family and logit link. All the models were adjusted for plaque index, abutment type, prosthesis type, and time with prosthesis in function (months). The site was added into the model for the site level analysis. Receiver operating characteristics (ROC) curve analysis was done to assess the cut‐point of MEA for predicting the BoP. The nature of this study was exploratory and no sample size calculation was conducted. Statistical testing was done within an exploratory framework at a two‐sided significance level of *α* = .05. All statistical tests were performed using Stata IC15 (StataCorp, 2017).

## 5 RESULTS

Forty‐seven patients (24 male and 23 female) with 103 bone level implant crowns in molar and premolar position were enrolled in the study (Table 1). Patients' average age was 61 years (range: 32–81) and average time with prosthesis in function was 39.17 months (SD 28.05, range 3–120 months). One patient was diagnosed with Diabetes Mellitus Type II under good metabolic control and 3 with hypertension also falling within the ASA type II Classification. Eighty‐eight crowns were screw retained and 15 cement retained. Crowns were either Porcelain‐fused‐to‐metal (PFM), monolithic Zirconia (all submucosal areas in glazed Zirconia), while 1 crown was non‐veneered made of Type III precious alloy (Table 1a).

**Table 1:** Descriptive data of 103 implants.

Implant Location	Mandible	Molars: 58
		Premolars: 13
	Maxilla	Molar: 22
		Premolar:10
Brand	Astra Tech	3 (2.91%)
	Straumann	100 (97.09%)
Diameter (mm)	3.6	1 (0.97%)
	4.1	29 (28.16%)
	4.2	2 (1.94%)
	4.8	71 (68.93%)
Length (mm)	8	95 (92.23%)
	9	3 (2.91%)
	10	5 (4.85%)

**Table 1a:** Retention and prosthesis types for 103 implant crowns.

	PFM	FMC	Zir	Total
Cement retained	7	0	8	15 (14.5%)
Screw retained	42	1	45	88 (85.5%)

Abbreviations: FMC, full metal crown; PFM, porcelain fused to metal; Zir, monolithic zirconia.

### 5.1 Site level analysis

Probing Depth measurement was deemed as not possible at 23 sites (3.72%). The average MEA for the excluded sites was 70.1° (range 67°−79°). The average Probing Depth was 2.82 mm (SD 0.90), while BoP was registered at 310 (50.16%) and Plaque at 330 (53.48%) sites respectively. The mean Probing Depth at mesial sites (buccal and lingual) 2.95 (SD 0.93) and at distal sites (buccal and lingual) 2.93 (0.94) was significantly higher than the directly buccal 2.57 (0.73) and directly lingual 2.59 (0.85) (*p* < .001) (One‐way repeated ANOVA, Table 2). The average MEA was 43.71° (SD 16.89), DA was 30.22° (SD 14.79) and TA 30.33° (SD 11.96). At molar sites the average MEA, DA and TA was 44.7°, 31.5° and 32.4°, while at premolars the same was 41.40°, 27.19°, and 25.6° respectively. The TA was the only feature that differed significantly between molars and premolars (*p* < .001).

**Table 2:** Measurements at site level (total and in each of six sites) with mean Probing Depth (PD) (mm and Standard Deviation in parenthesis), Bleeding and Plaque (number of positive sites and percentage in parenthesis).

	Total (*n* = 618)	MB	ML	BB	LL	DB	DL
PD Not measured	23 (3.72%)	5 (4.85%)	3 (2.91%)	3 (2.91%)	3 (2.91%)	3 (2.91%)	6 (5.83%)
PD measured	595 (96.28%)	98 (95.15%)	100 (97.09%)	100 (97.09%)	100 (97.09%)	100 (97.09%)	97 (94.17%)
Mean PD	2.82 (0.90)	2.98 (0.91)	2.92 (0.96)	2.57 (0.73)	2.59 (0.85)	2.96 (0.90)	2.90 (0.98)
		2.95 (0.93)^a^		2.57 (0.73)^a^	2.59 (0.85)^a^	2.93 (0.94)^a^	
Bleeding on probing	310 (50.16%)	48 (46.60%)	52 (50.49%)	48 (46.60%)	58 (56.31%)	49 (47.57%)	55 (53.40%)
		100 (48.54%)				104 (50.49%)	
Plaque presence	330 (53.48%)	48 (46.60%)	66 (64.08%)	40 (39.22%)	58 (56.31%)	48 (46.60%)	70 (67.96%)
		114 (55.34%)^b^				118 (57.28%)^b^	
Deep angle	30.2^c^ (14.79)	30.3^c^ (14.72)	31.3^c^(15.42)	29^c^ (13.34)	29.6^c^ (16.15)	31.2^c^ (15.24)	29.7^c^ (13.87)
		30.8^c^(15.05)				29.6^c^ (16.15)	
Mucosal angle	43.7^c^ (16.89)	44.3^c^ (17.58)	40.4^c^ (17.38)	44^c^ (17.09)	42.3^c^ (17.19)	46.1^c^ (15.04)	44.8^c^ (16.73)
		42.4^c^ (17.55)				42.4^c^ (17.19)	
Total angle	30.3^c^ (11.96)	32.2^c^ (10.47)	28.9^c^ (15.47)	31.6^c^ (10.64)	26.8^c^ (11.11)	32.6^c^ (10.44)	29.5^c^ (12.06)
		30.6^c^ (13.28)				26.9^c^ (11.11)	
Keratinised zone width	2.32 (1.61)	2.58 (1.57)		2.01 (1.54)		2.38 (1.68)	

Abbreviations: BB, Midbuccal; DB, Distobuccal, DL, Distolingual; LL, Midlingual; MB, Mesiobuccal, ML, Mesiolingual/palatal.

^a^ indicates one‐way repeated ANOVA *p* < .001.

^b^ indicates one‐way repeated ANOVA *p* < .05.

^c^ indicates degrees.

**Figure 3:**
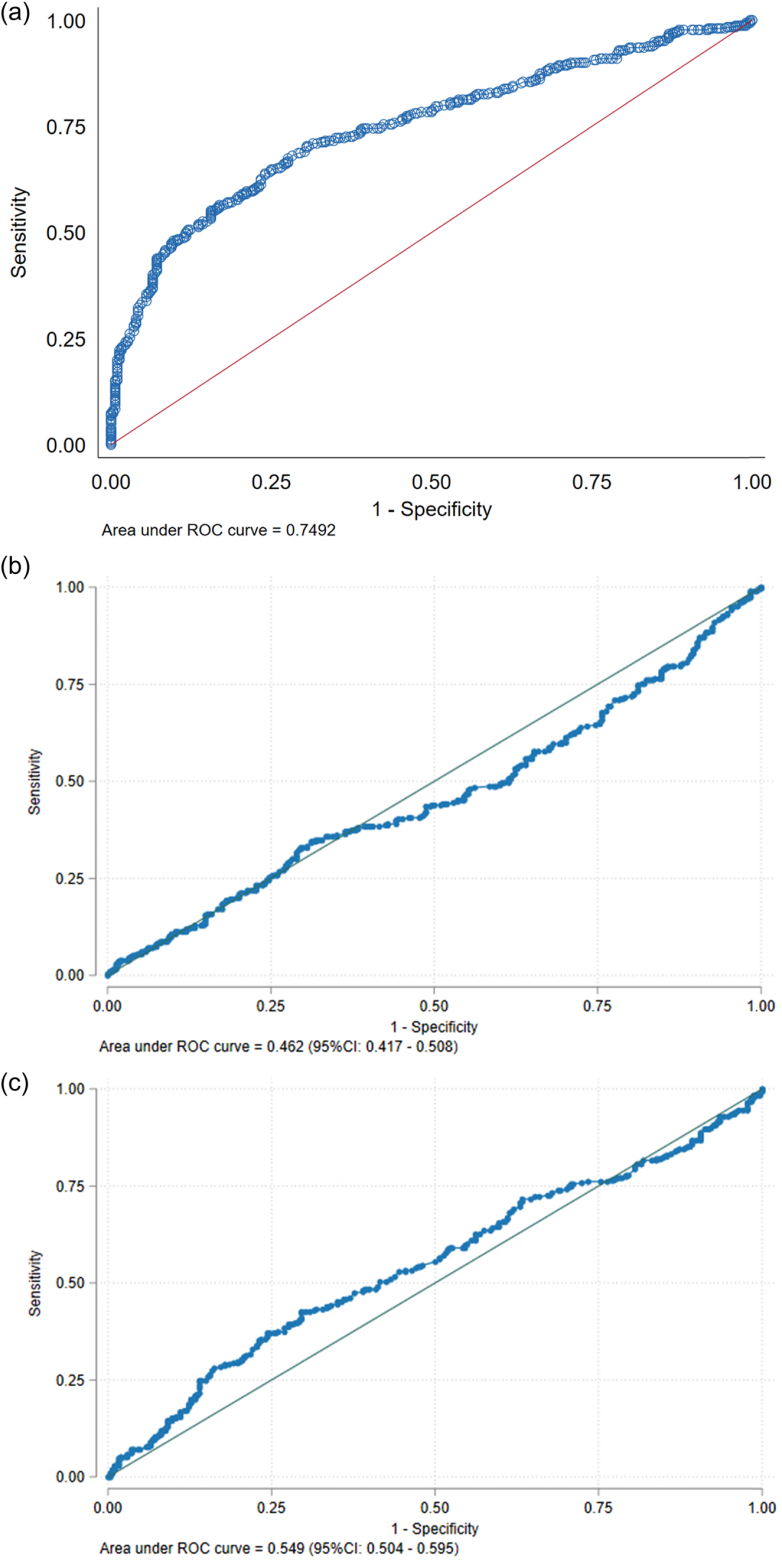
(a−c) Receiver operating characteristics (ROC) curve analysis for (a) Mucosal Emergence Angle (b) Deep Angle and (c) Total Contour Angle with Bleeding on Probing for a total of 618 sites.

After adjustment for site, plaque index, abutment type, prosthesis type and time with prosthesis in function (months), there was a significant correlation between MEA and BoP for all sites with an overall odds ratio of 1.07 (95% CI 1.05–1.09, *p* < 0.001) (Figure 3a). There was no association between specific site location and BoP (*p* = 0.383). The area under the curve of MEA to predict BoP was 0.749 (95% CI 0.711–0.788) (Figure 3a). Sites with MEA of more than or equal 30°, 40°, 50°, 60°, and 70° had a higher risk for BoP with an odds ratio of 3.16 (95% CI 1.88–5.33), 5.04 (95% CI 3.23–7.86), 7.597 (95% CI 4.83–13.17), 11.47 (95% CI: 5.99–21.99), and 33.55 (95% CI 7.13–157.89) respectively. No significant association was confirmed for DA and TA with BoP (Figure 3b,c. There was however a significant association between DA and TA (coefficient correlation = 0.45 (95% CI 0.38–0.53), *p* < 0.001) and MEA and TA (coefficient correlation = 0.37 (95% CI 0.26–0.48), *p* < 0.001).

### 5.2 Implant level analysis

**Figure 4:**
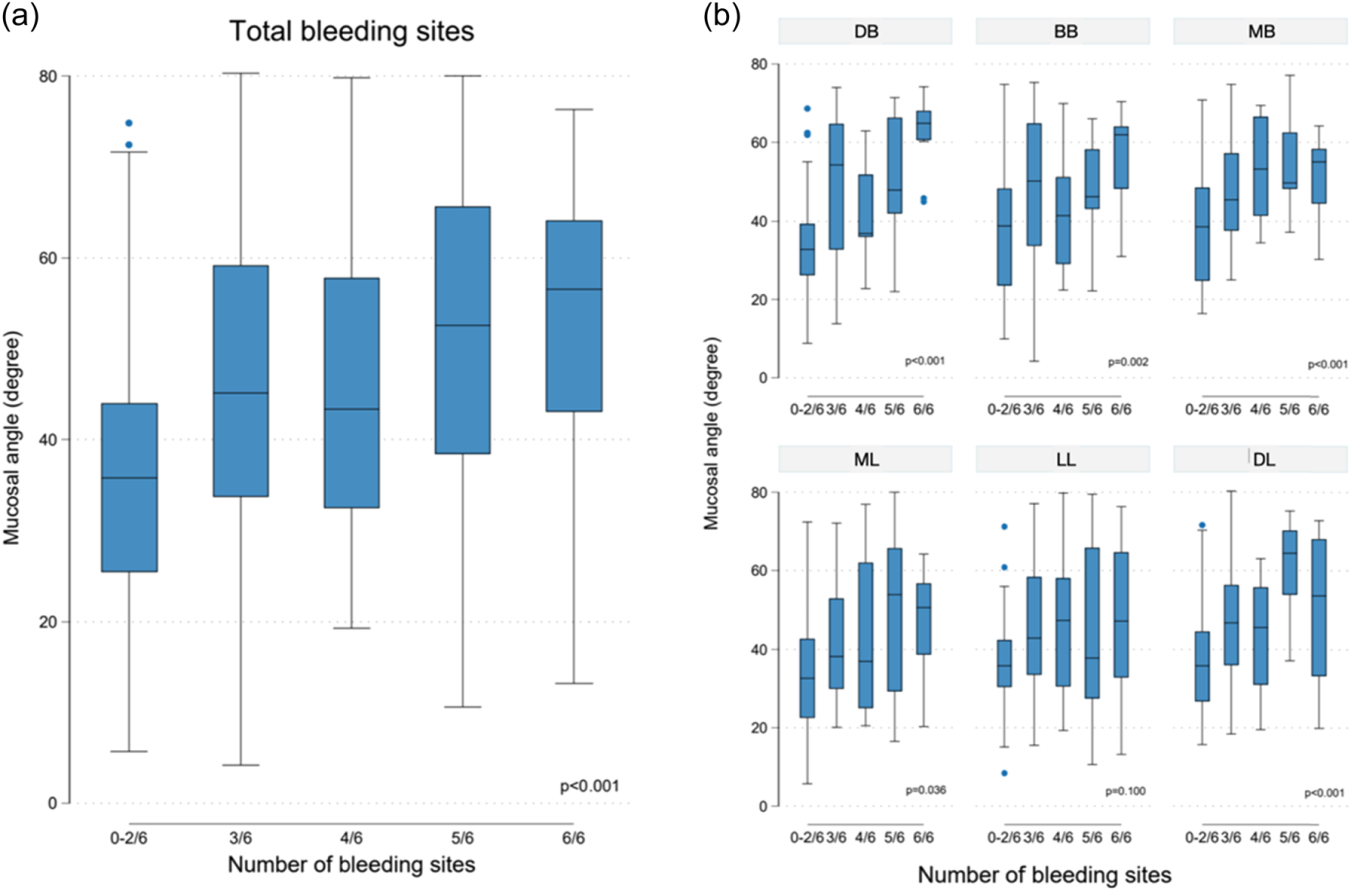
Box plot with the distribution of MEA values, outliers and median, for 5 different groups of implants as organised based to number of bleeding on probing sites out of 6 (0−2/6, 3/6, 4/6, 5/6, 6/6, (a) for the total number of sites and (b) for each of the 6 sites. MEA, Mucosal Emergence Angle.

Peri‐implant tissue inflammation at implant level (Vianna et al., 2018; Philip et al., 2022), is presented in Table 3 and Figure 4. Increase in the number of sites per implant harbouring MEA above all of the cut‐off points tested (≥30°, 40°, 50°, 60°) would significantly increase the number of bleeding sites as well (Table 4). When all 6 sites of an implant prostheses had MEA ≥ 40°, the risk of having bleeding at all 6 sites was 9.5 times higher (95% CI 1.70–52.97, *p* = 0.010).

**Table 3:** Prevalence of implants with mucositis (organised according to the number of bleeding on probing sites) and prevalence of implants with peri‐implantitis for the three case definitions.

		Implants (*n* = 103)
Mucositis	BoP+, 0−2 of 6 sites	37 (35.92%)
	BoP+, 3 of 6 sites	27 (26.21%)
	BoP+, 4 of 6 sites	14 (13.59%)
	BoP+, 5 of 6 sites	13 (12.62%)
	BoP+, 6 of 6 sites	12 (11.65%)
Peri‐implantitis 1	Bop, PD ≥ 6, BL ≥ 3	2 (1.9%)
Peri‐implantitis 2	BoP, BL ≥ 3 mm	8 (7.77%)
Peri‐implantitis 3	BoP, BL ≥ 2 mm	10 (9.71%)

Abbreviations: BL, bone loss; BoP, bleeding on probing.

**Table 4:** Association between number of sites with angles above each cut‐point and number of bleeding on probing sites per implant (column A) and the two case definitions of Peri‐implantitis (columns B, C) (implant level analysis).

Angle cut‐off point	A. Mucositis^a^		B. Peri‐implantitis 2^b^		C. Peri‐implantitis 3^b^	
			(BoP, BL ≥ 3 mm)		(BoP, BL ≥ 2 mm)	
	Adjusted IRR (95% CI)	*p*	Adjusted OR (95% CI)	*p*	Adjusted OR (95% CI)	*p*
MEA						
≥30°	1.12 (1.02–1.23)	0.016	0.75 (0.30–1.87)	0.537	1.07 (0.47–2.43)	0.867
≥40°	1.15 (1.07–1.24)	<0.001	1.63 (0.70–3.81)	0.260	1.69 (0.75–3.81)	0.209
≥50°	1.15 (1.07–1.23)	<0.001	1.27 (0.64–2.54)	0.494	1.37 (0.66–2.83)	0.396
≥60°	1.12 (1.04–1.21)	0.004	1.60 (0.75–3.42)	0.227	1.71 (0.74–3.94)	0.210
DA						
≥30°	0.98 (0.93–1.03)	0.450	1.02 (0.56–1.85)	0.952	1.06 (0.62–1.81)	0.831
≥40°	1.01 (0.95–1.08)	0.713	0.97 (0.47–1.97)	0.924	0.79 (0.30–2.05)	0.629
≥50°	1.02 (0.92–1.12)	0.752	1.58 (0.80–3.11)	0.190	0.92 (0.34–2.52)	0.877
≥60°	1.01 (0.87–1.16)	0.943	1.66 (0.60–4.58)	0.329	1.32 (0.37–4.73)	0.666
TA						
≥30°	1.05 (0.98–1.12)	0.190	1.48 (0.73–3.02)	0.279	1.56 (0.75–3.27)	0.237
≥40°	1.03 (0.94–1.12)	0.541	1.34 (0.61–2.92)	0.463	1.36 (0.63–2.96)	0.433
≥50°	1.05 (0.92–1.19)	0.477	2.11 (0.59–7.54)	0.253	1.19 (0.26–5.51)	0.824
≥60°	1.05 (0.83–1.33)	0.663	1.58 (0.28–8.94)	0.607	1.00 (0.09–11.15)	1.000

*Note*: All the models were adjusted for number of positive plaque index sites, abutment, prosthesis, and time with prosthesis in function (months) for each angle cut‐off point.

^a^ The estimated incidence rate ratio (IRR) of mucositits were done by generalized estimating equations (GEE) with poisson family and log link, accounting for multiple implants within patient;

^b^ For peri‐implantitis, the estimated adjusted odds ratio (OR) was performed using GEE with binomial family and logit link.

The prevalence of peri‐implantitis at implant level (103 implant) for the three case definitions used (PD ≥ 6 and BL ≥ 3, BL ≥ 3 mm, and BL ≥ 2 mm was 2 (1.9%), 8 (7.8%) and 10 (9.7%) respectively (Table 3). There was no association between the diagnosis of peri‐implantitis (case definitions 1, 2, and 3) and all angles (MEA, DA, and TA), although the results suggest that the more sites with angles 40°, 50°, and 60°, the greater the risk of peri‐implantitis (Table 4).

## 6 DISCUSSION

The results of this study suggest that the angle of the prosthesis at the point of emergence from the peri‐implant mucosa can be significantly associated with the prevalence and extend of inflammation. Attempting to identify a specific threshold for the value of the MEA might be statistically possible, but in terms of clinical relevance the results of this study suggest to maintain this angle as narrow as possible. The protocol devised for this study aimed to build on previous research, while utilising 3D imaging to increase precision in defining and measuring the respective structures. 3D imaging was employed to identify representative vertical and horizontal planes of the Implant Supracrestal Complex (Mattheos, Vergoulis, et al., 2021), while accounting for the level of the soft tissue and thus the exact points of emergence. This way it introduced precisely defined and reproducible reference points for the measurement of angles.

Three angles were defined and measured, aiming to represent different aspects of the Implant Supracrestal Complex. The MEA represented the angle of the prosthesis at the exact point of emergence from the peri‐implant mucosa, as described by the Yotnuengnit et al. (2008) and the 9th edition of the Glossary of Prosthodontic Terms. This is the critical interface where bacterial plaque would first come in contact with the peri‐implant mucosa.

The DA on the other hand describes the relation of the abutment as it ascends directly from the bone level. This angle has been shown to influence early marginal bone loss (Souza et al., 2018). The TA was defined in a similar manner as in previous studies on periapical radiographs and represented a rough approximation of the total contour, which also served as a “control,” to relate these results to previous work (Katafuchi et al., 2018; Yi et al., 2020).

Unlike previous studies however (Yotnuengnit et al., 2008), a tangent line was rejected for the calculation of the EA. A tangent line can only be reproducibly defined in a specific point in the perimeter of a perfect circle. In the diverse convexity/concavity of the prosthesis contour, defining a tangent line comes inevitably with a high level of subjectivity. This could result to limited reproducibility and clinical relevance of such measurements. Therefore, two specific points at respective parallel planes were identified to define each angle. The two planes were 0.5 mm apart in the case of the MEA to define the angle of the prosthesis contour at the exact point of emergence through the peri‐implant mucosa. The DA, representing the establishment of the peri‐implant “biologic width” (Glauser et al., 2005), was defined by two planes of 1.5 mm distance. Finally, the TA was defined as in periapical radiograph studies with one point set at the bone level and the other arbitrarily at the point of maximum convexity of the prosthesis contour.

The present study showed a clear association of the MEA and peri‐implant tissue inflammation. The risk for bleeding at site level increases significantly for wider values of the MEA with the odds ratio from 1 for angle of 10°, to more than 33 for angle wider than 70°. Although a statistical “cut off” threshold might be possible to define arbitrarily, such a threshold might be more related to the specific sample rather than a biological imperative. At implant level, a drastic increase in risk for inflammation appeared when all 6 sites of the prostheses had MEA ≥ 40°, thus this threshold was highlighted as indicative. In clinical terms the recommendation should be to design prosthesis with as narrow MEA as feasible and certainly avoiding wide angles of more than 40°.

Despite this clear association, the MEA was not significantly correlated with peri‐implantitis in this sample. The short observation period (39 months) of this patient sample might be a reason for this observation. A meta‐regression performed by Derks and Tomasi (2015) on 11 studies on the prevalence of peri‐implant mucositis and peri‐implantitis, demonstrated a positive association between function time and the prevalence of peri‐implantitis (Derks & Tomasi, 2015). Nevertheless, consensus that mucositis is a precedent of peri‐implantitis the onset of which may occur early during follow‐up and follow a nonlinear progression (Schwarz et al., 2018). Thus, it is not uncommon for cross sectional studies aiming to report the prevalence of peri implant diseases after mean observation periods of 22–42 months (Schwarz et al., 2017; Maximo et al., 2008; Ferreira et al., 2006; Matarazzo et al., 2018). In particular when the aim is to study outcomes related to Mucositis studies have used observation periods of 6−24 months (de Tapia et al., 2019; Boynuegri et al., 2013; Vianna et al., 2018).

Previous studies have shown association between angles of the prosthesis and peri‐implantitis, but the observation period in these cases were 5 (Yi et al., 2020) and 10.9 years (Katafuchi et al., 2018). Furthermore, the two aforementioned studies utilised a line drawn in periapical radiographs to define an overall “contour” angle, irrespective of the peri‐implant tissue margin. The total angle (TA) in this study was significantly correlated with the DA, which implies that the total contour can be possibly used as a surrogate of the DA. This design feature might be an important parametre in the comprehension of previous findings.

**Figure 5:**
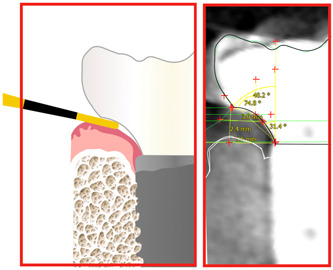
Radiographic image (right) and scematic representation (left) of an actual site where probing depth measurement was deemed as unrealiable, as the overcontouring prevented the appropriate insertion of the probe. The respective MEA at this site was 74.8°. MEA, Mucosal Emergence Angle.

The plaque‐induced inflammatory diseases are first manifested as a reversible, marginal tissue inflammation at the coronal margin of the peri‐implant soft tissue (Salvi et al., 2022). Experimental studies in humans have documented the causal role of plaque accumulation for the development of peri‐implant mucositis (Pontoriero et al., 1994, Zitzmann et al., 2001), while the 2017 World Workshop on the Classification of Periodontal and Peri‐Implant Diseases and Conditions (Berglundh et al., 2018) has accepted Peri‐implant Mucositis being a precursor to Peri‐implantitis based on evidence from observational studies. It is at present well established that patients with poor plaque control are at higher risk of developing peri‐implantitis. As plaque accumulates on the prosthesis, the impact of plaque will be first manifested on the peri‐implant mucosal margin. Thus, the original concept of EA as described by Yotnuengnit et al. (2008) focused on the angle of the prosthesis at the point of emergence, as this was seen as a critical element for plaque retention and consequent gingival inflammation. The results of this study showed that a wide angle of the prosthesis at the point it emerges through the mucosal margin could lead to mucositis. This did not appear to be related to the total contour or the DA. The relatively low prevalence of peri‐implantitis in this sample combined with short observation period of 39 months on average does not allow any conclusions with regard to the potential role of prosthetic angles in peri‐implantitis. Further research and in particular prospective studies will be required to better understand these findings. All three case definitions for peri‐implantitis included interproximal bone loss, as measured in periapical radiographs. Reporting and analysing of radiographic marginal bone level was beyond the scope of this study. However, utilising measurements on periapical radiographs for the sole purpose of identifying peri‐implantitis cases was deemed essential for consistency with previously published studies, as well as to comply with established case definitions. In the future, the case definitions of peri‐implantitis might be improved with utilisation of 3D‐imaging. Finally, the diagnostic use of probing is not without important limitations. Although it is well established that over contouring of the implant prosthesis can limit access to probing and reliable measurement of probing depth (Salvi et al., 2022; Garcia‐Garcia et al., 2021), there is no current consensus practice of how this issue should be addressed in research. As this limitation results exclusively to underestimation of probing depth, ignoring this problem can risk potentially seriously compromising an analysis (Figure 5). The authors have therefore excluded from the analysis all sites where the contour prohibited appropriate insertion of the probe based on the clinical judgement of the examiner, which was less than 4% of the sites. Interestingly, the average MEA at these sites was 70.1°, offering a good indication of the level of over contouring that becomes a hindrance to essential diagnostic procedures. Despite the inevitable degree of subjectivity in the clinical judgement, the authors believe that knowingly including underestimated probing depth measurements is a far bigger threat to the validity of the analysis than a structured attempt to exclude them, albeit imperfect. The problem of probing depth measurements around over contoured implant prostheses and how this could influence our diagnosis and in particular research analyses might be a topic to be addressed by future consensus.

The three vertical planes for the measurements were selected on the basis of representing different parts of the prosthesis design, as the mesiodistal and buccolingual profiles of restorations might be very different to the buccal and palatal ones. One further consideration was to assess sites where probing depth and BoP can be directly correlated with the prosthesis shape. The mesiodistal vertical plane (as seen in periapical radiographs) was not utilised, since the peri‐implant sulcus is not accessible for probing at these sites when neighbouring teeth are present. In particular at molar sites, the nearest point accessible to probing can be 5 mm or more away from the point where the prosthesis contour is assessed, and the marginal bone loss is measured in periapical radiographs.

A margin of error to a certain extent is inevitable in all operator‐conducted measurements. Nevertheless, by reducing the degree of subjectivity in defining the key points for measurements, this method could reach higher level of reproducibility than previous attempts based on tangent lines on radiographs. A software built for precise 3D measurements was used, while a high level of interrater agreement was reached, suggesting good reproducibility of the results.

The implants included in this study were all bone level placed at posterior sites, aiming to reduce the heterogeneity of the sample. Statistical models were utilised to account for most common but not all possible confounding factors. Thus factors such as plaque, time with prosthesis, prosthesis and abutment type were accounted for. Other factors such as patient age, patient periodontal disease status, smoking status, implant diameter, implant type were not accounted for in the analysis. Larger studies in the past have not found any association of the latter group of factors with the studied parametres (Katafuchi et al., 2018). None of the patients in this study was diagnosed with Diabetes, but certain confounding factors such as implant depth and concavity/convexity might have been interesting to account for in future studies. Convexity of the prosthesis contour has been in the past being associated with marginal bone loss (Valente et al., 2020), recession (Siegenthaler et al., 2022) and peri implantitis (Yi et al., 2020). Nevertheless, the 3D analysis of the EA has shown that a large number of cases cannot fit within a dichotomous assessment of convexity/concavity, as areas of convexity are followed by areas on concavity in the same contour. Future studies might have to develop a more robust assessment model for convexity, to reflect the entire spectrum of complexity of the transmucosal contour. Other parametres, such as implant‐abutment and abutment‐prosthesis interfaces in relation to the tissue dimensions have not been included in this analysis, but could be important parametres to investigate in future studies. All implants in this study were placed, restored and maintained by postgraduate residents and staff in two postgraduate university clinics. The prosthetic, surgical and maintenance protocols followed have adhered to the current best practices and university standards. The sample utilised in this study might not be characteristic of all diverse prosthetic designs that can be encountered in the wider implant patient population.

The results of this study, have to been seen in light of the limitations of the methodology. The statistical analysis accounted for the most common, but not all potential confounding factors. Approximately 4% of the probing depth measurements were excluded from the analysis based on the clinical judgement of the examiner. The observation period might be not adequate to study peri‐implantitis. Mucositis has been described based on the extent of dichotomous BoP, as by current established practice in epidemiological research and clinical trials (de Tapia et al., 2019; Vianna et al., 2018). The authors acknowledge that the use of an implant‐based case definition of mucositis would have been desirable, which however would require a staged BoP index (e.g. Gingival Index by Loe & Silness), as well as some classification based on tissue morphology to be applied by calibrated examiners at the time of clinical examination as suggested by the case definition described by Renvert et al. (2018) for “day to day clinical practice.” Such a collective instrument has not been utilized for research as of this point.

Conclusively, these results have shown a significant correlation between the MEA and peri‐implant tissue inflammation as defined by BoP at both site and implant level. Maintaining an angle no wider than 30°−40° at the point of emergence of the prosthesis is advisable, while the aim should be to keep this angle as narrow as clinically feasible.

## AUTHOR CONTRIBUTIONS

*Conceptualization*: Martin Janda, Nikos Mattheos, Mansuang Arksornnukit, Wareerat Chengprapakorn. *Data curation*: Nikos Mattheos, Mansuang Arksornnukit, Wareerat Chengprapakorn. *Formal Analysis*: Nikos Mattheos, Mansuang Arksornnukit. *Funding acquisition*: Nikos Mattheos. *Investigation*: Piboon Rungtanakiat, Natchaya Thitaphanich, Nikos Mattheos. *Methodology*: Martin Janda, Nikos Mattheos, Mansuang Arksornnukit. *Project administration*: Mansuang Arksornnukit, Nikos Mattheos, Wareerat Chengprapakorn. *Resources*: Nikos Mattheos, Mansuang Arksornnukit, Wareerat Chengprapakorn. *Software*: Piboon Rungtanakiat, Natchaya Thitaphanich. *Supervision*: Nikos Mattheos, Mansuang Arksornnukit. *Validation*: Nikos Mattheos, Piboon Rungtanakiat, Natchaya Thitaphanich. *Visualization*: Martin Janda, Nikos Mattheos. *Writing*—*original draft*: Nikos Mattheos, Piboon Rungtanakiat. *Writing*—*review and editing*: Martin Janda, Nikos Mattheos, Wareerat Chengprapakorn, Mansuang Arksornnukit.

## CONFLICT OF INTEREST STATEMENT

The authors declare no conflict of interest.

## Data Availability

Data is available from corresponding author upon reasonable request
